# Prevalence of thyroid dysfunction and associated factors among adult type 2 diabetes mellitus patients, 2000–2022: a systematic review and meta-analysis

**DOI:** 10.1186/s13643-024-02527-y

**Published:** 2024-04-30

**Authors:** Rishan Hadgu, Abebaw Worede, Sintayehu Ambachew

**Affiliations:** 1 Department of Medical Laboratory Science, College of Medicine and Health Sciences, Arba Minch University, P.O. Box 21, Arba Minch, Ethiopia; 2 Department of Clinical Chemistry, School of Biomedical and Laboratory Sciences, College of Medicine and Health Sciences, University of Gondar, P.O. Box 196, Gondar, Ethiopia; 3 Adelaide Medical School, University of Adelaide, Adelaide, SA Australia

**Keywords:** Thyroid Dysfunction, Type two Diabetes mellitus, Factors, Meta analysis, Systematic review

## Abstract

**Background:**

Thyroid dysfunction (TD) and type 2 diabetes mellitus (T2DM) frequently co-occur and have overlapping pathologies, and their risk increases with age. Thyroid dysfunction along with T2DM will worsen macro- and microvascular complications, morbidity, and mortality.

**Methods:**

The Preferred Reporting Items for Systematic Reviews and Meta-Analyses statement guideline was followed. The databases used were Embase, ScienceDirect, PubMed, and Google Scholar. The Joana Briggs Institute (JBI) scale was used to assess the quality of the included studies. The data was extracted by Microsoft Excel and analyzed through STATA version 14 software. The overall pooled prevalence of TD and its main components were estimated using the random-effects model. The consistency of studies was assessed by *I*^2^ test statistics. Pooled meta-logistic regression was used to present the pooled prevalence with a 95% confidence interval (CI). Besides, subgroup and sensitivity analyses were employed.

**Result:**

Thirty-eight studies were included. The pooled prevalence of TD was 20.24% (95% *CI*: 17.85, 22.64). The pooled prevalence of subclinical hypothyroidism, hypothyroidism, subclinical hyperthyroidism, and hyperthyroidism was found to be 11.87% (95% *CI*: 6.90, 16.84), 7.75% (95% *CI*: 5.71, 9.79), 2.49% (95% *CI*: 0.73, 4.25), and 2.51% (95% *CI*: 1.89, 3.13), respectively. Subgroup analysis based on continent revealed a higher prevalence of TD in Asia and Africa. Factors like being female, *HbA1c* ≥ 7%, DM duration > 5 years, family history of TD, central obesity, smoking, the presence of retinopathy, and neuropathy were found associated with TD.

**Conclusion:**

The current systematic review and meta-analysis showed that the TD’s pooled prevalence was relatively higher than the general population. Therefore, regular screening of TD should be done for T2DM patients.

## Introduction

Thyroid dysfunction (TD), the most common endocrinal pathology next to diabetes mellitus (DM) [1], is a condition characterized by an increased or decreased production of thyroid hormones (TH) [2]. TDs occur as hypothyroidism (clinical or subclinical) or hyperthyroidism (clinical or subclinical) and are reflected in circulating levels of free triiodothyronine (FT3), free thyroxine (FT4), and TSH [3]. Type 2 diabetes mellitus (T2DM) is characterized by hyperglycemia as a result of insulin resistance and impaired pancreatic beta-cell activity [4, 5]. Obesity, a sedentary lifestyle, energy-dense foods, smoking, alcohol intake, and population aging are the key risk factors for T2DM [6].

Type 2 diabetes mellitus and TD often co-occur and have overlapping pathologies, and their risk increases with age. TDs are significantly more prevalent among T2DM patients [1]. TDs affect approximately 10 to 15% of the patients with diabetes, whereas in non-diabetes, the prevalence is approximately 6% [3]. The prevalence of TD in T2DM varies between studies, ranging from very low (5.5%) to very high (75%) [7]. Furthermore, studies have also recorded a higher prevalence of TD (31.4%) among females with T2DM [8]. Subclinical hypothyroidism is the most common type of TD among the diabetic population [9–11].

There is a complex relationship between TD and DM that has yet to be discovered. The pathophysiological link between T2DM and TD is thought to be the outcome of a complex interaction of biochemical, genetic, and hormonal abnormalities [12]. T2DM influences the TH in two sites, first at the level of hypothalamus by controlling TRH release and second at the peripheral tissues by impairing the conversion of T4 to T3 [13, 14]. The hypothalamus–pituitary–thyroid axis may be disrupted by experimentally induced diabetes, which lowers plasma TRH and TSH levels, lowering TH synthesis. [15]. In addition to this, anti-diabetics such as sulfonylureas and thiazolidinedione group drugs (e.g., pioglitazone) can negatively impact thyroid function [12].

Thyroid dysfunction can also cause T2DM. Both hypothyroidism and hyperthyroidism have been investigated to be associated with DM [1]. Hypothyroidism is associated with reduced glucose absorption from GIT, and it is accompanied by prolonged peripheral glucose accumulation, diminished hepatic glucose output, and reduced utilization of glucose, which were considered hallmarks of diabetes [16]. On the other hand, hyperthyroidism promotes hyperglycemia, and several theories have been proposed to explain this impact. In a thyrotoxic environment, the half-life of insulin is shortened, which is assumed to be related to the accelerated degradation of the active hormone and the release of inactive precursors [17]. In addition, hyperthyroidism is also hypothesized to boost glucose production through a variety of processes, including upregulation of gluconeogenesis as a result of increased lipolysis and lactate overproduction, as well as increased hepatic output due to increased expression of the GLUT2 glucose transporter [18].

The coexistence of TD in T2DM patients will worsen the macro-vascular and microvascular complications, morbidity, mortality, and quality of life [11]. Evidence indicates that subclinical hypothyroidism compromises both micro- and macrovascular function, increasing the risk of peripheral neuropathy, peripheral artery disease, and diabetic nephropathy [13, 19]. In addition to this, both subclinical hyperthyroidism and T2DM have been associated with an increase in cardiovascular disease risk and mortality [20]. Both TD and DM, especially uncontrolled diabetes, cause many health problems. Increased frequency of hypoglycemia in hypothyroidism and development of potentially life-threatening ketoacidosis in thyrotoxicosis are the most serious effects [21].

Detecting TD in T2DM patients would help clinicians provide the best treatment for metabolic problems, as TDs like hypothyroidism can make achieving a glycemic target and managing other comorbidities difficult [11]. Screening of TD, especially the subclinical dysfunction, in patients with DM is justified because most patients can be asymptomatic [22]. The strong link between diabetes and TD encouraged the American Diabetes Association to propose that people with diabetes must be checked periodically for TD [23].

There are different studies conducted to assess the prevalence and associated factors of TD among T2DM all over the world. Despite their results having a great disparity and inconsistent findings, moreover, there is no previous systematic review and meta-analysis that estimated the prevalence and associated factors of TD among T2DM. Therefore, the current systematic review and meta-analysis is designed to assess the pooled prevalence and associated factors of TD among T2DM patients.

## Methods and materials

### Eligibility criteria

#### Inclusion criteria

Studies on the prevalence and associated factors of TD among adult T2DM patients published in different peer-reviewed journals between 2000 and 2022 were included. All studies were original research published in English and contained the minimum information concerning sample size and status of TD, which helped to analyze a pooled estimate of the prevalence of TD and associated factors among adult T2DM patients. Besides, studies in which TD has been classified as hypothyroidism, hyperthyroidism, subclinical hypothyroidism, and subclinical hyperthyroidism using laboratory measurements of TSH, T4, and T3 were included.

Hypothyroidism is characterized by elevated serum TSH levels, a low serum FT4 level, and low FT3 [24, 25], whereas hyperthyroidism is characterized by elevated serum FT4 and FT3 and low levels of TSH levels [26]. Subclinical hyperthyroidism is characterized by decreased serum TSH concentration in association with a normal serum FT4 and FT3 concentrations [26]. Subclinical hypothyroidism is defined as an elevated serum TSH level associated with normal total or FT4 and FT3 levels [20]. Studies that used International Diabetes Federation (IDF) criteria for diagnosing T2DM were included. The IDF criteria state diagnostic criteria for diabetes which is maintained fasting plasma glucose ≥ 7.0 mmol/l (126 mg/dl) or 2–h plasma glucose ≥ 11.1 mmol/l (200 mg/dl) [27].

#### Exclusion criteria

Articles written in another language other than English were excluded. Studies conducted among type 1 DM patients, and diabetic neuropathy patients, were excluded. Studies from non-original papers (literature reviews, books) were also excluded. Irrelevant and duplicated papers were excluded. Articles which lacked necessary information such as age and year of study were also excluded. Studies that did not show the diagnostic criteria for both T2DM and TD were omitted. Furthermore, articles that did not provide information on the overall prevalence of TD were omitted.

### Search strategy

**Fig. 1:**
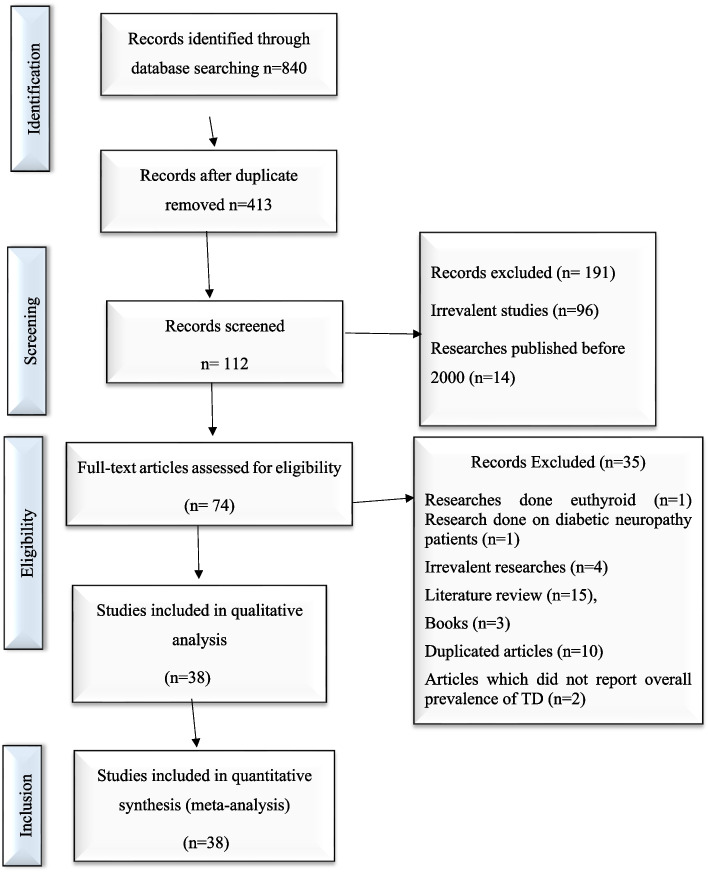
Flow chart to describe the selection of studies for the systematic review and meta-analysis of the prevalence of TD and associated factors among adult type 2 DM patients

The Preferred Reporting Items for Systematic Reviews and Meta-Analyses (PRISMA) statement guideline was used to report this systematic review and meta-analysis [28]. An electronic search was conducted to retrieve studies. Published articles of cross-sectional, case–control, cohort, prospective, case series, and retrospective studies were included. Embase, PubMed, Google Scholar, and ScienceDirect literature were the electronic databases used to identify studies conducted on the prevalence of TD and associated factors among T2DM patients published from 2000 to 2022. The search terms were used in agreement with the title/abstract using the arrangement of keywords that were used to select relevant studies. Figure 1 shows the flow chart used to describe the selection of studies.

Using Boolean operators like “OR” and “AND,” the search terms were utilized separately and in combination. An example of the search strategy used to retrieve relevant articles was as follows: (((((((prevalence[Title/Abstract]) AND (hypothyroidism[Title/Abstract])) AND (hyperthyroidism [Title/Abstract])) AND (thyroid disorders[Title/Abstract])) OR (thyroid dysfunction [Title/Abstract])) AND (adult[Title/Abstract])) AND (type 2 diabetes mellitus[Title/Abstract])) OR (insulin resistant diabetes[Title/Abstract]). Duplicated data were excluded. The software EndNote version X8 (Thomson Reuters, New York, NY, USA) was used to manage references and remove duplicated references.

### Search method and quality assessment

An electronic search was conducted in Embase, PubMed, Google Scholar, and ScienceDirect literature using the keywords to include articles that were published from 2000 to 2022. Then, searched articles were screened by the title and abstract to consider the articles in the full-text review. Following the exclusion of duplicates, the abstracts and titles of 413 papers were screened for eligibility criteria, and 38 were chosen for full-text evaluation.

This systematic review and meta-analysis is based on original research articles. For maintaining the quality of the review, all duplications were checked thoroughly. The abstracts of these articles were checked deeply for the analysis and purification. A careful evaluation of each research paper was carried out at later stage.

The quality of the studies was assessed using the Joana Briggs Institute (JBI) standardized critical appraisal instrument for prevalence studies scale. The following items were used to appraise the included studies: (Q1) Was the sample frame appropriate to address the target population?, (Q2): Were study participants sampled in an appropriate way?, (Q3): Was the sample size adequate?, (Q4): Were the study subjects and the setting described in detail?, (Q5): Was the data analysis conducted with sufficient coverage of the identified sample?, (Q6): Were valid methods used for the identification of the condition?, (Q7): Was the condition measured in a standard, reliable way for all participants?, (Q8): Was there appropriate statistical analysis?, and (Q9): Was the response rate adequate, and if not, was the low response rate managed appropriately? Table 1 shows the methodological quality assessment of included studies using the Joana Briggs Institute (JBI) standardized critical appraisal instrument for prevalence studies scale.

**Table 1:** Methodological quality assessment of included studies using Joana Briggs Institute (JBI) standardized critical appraisal instrument for prevalence studies

First author/year	Q1	Q2	Q3	Q4	Q5	Q6	Q7	Q8	Q9	Total
Zhu, Y. et al. (2019) [26]	N	N	Y	Y	Y	Y	Y	Y	Y	7
Vamshidhar et al. (2020) [16]	Y	Y	N	Y	Y	Y	Y	Y	Y	8
Subekti, I. et al. (2017) [11]	Y	Y	Y	Y	Y	Y	Y	Y	Y	9
Papazafiropoulou, A. et al. (2010) [27]	Y	Y	Y	Y	Y	Y	N	Y	N	7
Palma, C. C. et al. (2013) [22]	Y	Y	Y	Y	Y	Y	Y	Y	Y	9
Ogbonna, S. U. and I. U. Ezeani (2019) [28]	Y	Y	Y	Y	Y	Y	Y	Y	Y	9
Khatiwada, S. et al. (2015) [29]	Y	Y	Y	N	Y	Y	Y	Y	Y	8
Díez, J. J. et al. (2012) [20]	Y	Y	Y	Y	Y	Y	Y	Y	Y	9
Al-Geffari, M. et al. (2013) [23]	Y	Y	Y	Y	Y	Y	Y	Y	Y	9
Radaideh, A. et al. (2004) [30]	Y	Y	Y	N	Y	Y	Y	Y	Y	8
Ozair, M. et al. (2021) [31]	Y	Y	Y	Y	Y	Y	Y	Y	Y	9
Elgazar, E. H. et al. (2019) [32]	Y	Y	Y	Y	Y	Y	Y	Y	Y	9
Moslem, F. et al. (2015) [14]	Y	Y	Y	Y	N	Y	Y	Y	Y	8
Younes Y. R. (2020) [33]	Y	Y	Y	Y	Y	Y	Y	Y	Y	9
Haque S. I. U. et al. (2019) [34]	Y	Y	Y	Y	Y	Y	Y	Y	Y	9
Ahmed A. A. et al. (2017) [3]	Y	Y	Y	Y	Y	Y	Y	Y	Y	9
Papanna J. et al. (2019) [8]	N	Y	Y	N	Y	Y	Y	Y	Y	8
Mausam Jain et al. (2020) [35]	Y	Y	Y	Y	N	Y	Y	Y	Y	8
Al-Wazzan, H. et al. (2010) [36]	Y	Y	Y	Y	Y	Y	Y	Y	Y	9
Jain G. et al. (2013) [37]	N	Y	N	Y	N	Y	Y	Y	Y	6
Noori D. A. S. et al. (2019) [38]	Y	N	Y	Y	Y	N	Y	Y	Y	7
Jalal M. J. et al. (2019) [39]	Y	Y	N	Y	Y	Y	N	Y	Y	7
Ghazali et al. (2010) [40]	Y	Y	Y	N	Y	N	Y	Y	Y	7
Vikhe, V. B. et al. (2013) [9]	Y	Y	Y	Y	N	N	Y	Y	Y	7
Akbar D. H. et al. (2006) [41]	N	Y	Y	Y	N	Y	Y	Y	Y	7
Khassawneh, A. H. et al. (2020) [42]	Y	Y	Y	N	Y	Y	N	Y	Y	7
Sarfo-Kantanka O. et al. (2017) [43]	Y	Y	Y	N	N	Y	Y	Y	Y	7
Padma V. et al. (2015) [44]	N	Y	N	Y	Y	Y	Y	Y	Y	7
Hussain T. et al. (2019) [45]	N	Y	N	Y	Y	Y	Y	Y	Y	7
Peters, K. E. et al. (2020) [46]	Y	Y	Y	N	Y	Y	Y	Y	Y	8
Yadav, A. et al. (2021) [47]	Y	Y	Y	N	Y	Y	Y	Y	Y	8
Tudor, R. M. et al. (2018) [10]	Y	Y	Y	Y	Y	Y	Y	Y	Y	9
Demitrost, L. et al. (2012) [48]	Y	Y	Y	Y	Y	Y	Y	Y	Y	9
Elmenshawi I. et al. (2017) [49]	Y	Y	Y	N	Y	Y	Y	Y	Y	8
Al-Lehibi K. I. et al. (2019) [50]	Y	Y	Y	Y	Y	N	Y	N	Y	7
Jugati A., Biradar M. (2018) [51]	Y	Y	Y	Y	Y	Y	Y	Y	Y	9
Aljabri K. S. (2019) [52]	Y	Y	Y	Y	Y	Y	Y	Y	Y	9
Vij V. et al. (2014) [53]	Y	Y	Y	N	Y	N	Y	Y	Y	7

Q1: Was the sample frame appropriate to address the target population? Q2: Were study participants sampled in an appropriate way? Q3: Was the sample size adequate? Q4: Were the study subjects and the setting described in detail? Q5: Was the data analysis conducted with sufficient coverage of the identified sample? Q6: Were valid methods used for the identification of the condition? Q7: Was the condition measured in a standard, reliable way for all participants? Q8: Was there appropriate statistical analysis? Q9: Was the response rate adequate, and if not, was the low response rate managed appropriately?

*Y* yes, *N* no, *U* unclear

### Data extraction

An established data extraction tool, Microsoft Excel 2013 spreadsheet, was used for the data extraction. Three authors (R. H., A. W., and S. A.) independently conducted a search in Embase, PubMed, Google Scholar, and ScienceDirect databases. This tool extracted information such as the author’s name, publication year, study design, sample size, prevalence of TD, prevalence of subgroups of TD, and the laboratory diagnostic method used to diagnose TD, and T2DM were all extracted using this tool. PRISMA guideline was strictly followed when conducting this review.

### Data processing and analysis

Data was entered and analyzed using STATA version 14 after extracting the data from all eligible studies. Overall, pooled prevalence of TD and its main components were estimated using the random-effects model. In the meta-analysis, to assess the consistency of studies, *I*^2^ test statistics was used. This test examines the hypothesis of all the included studies is evaluated for the same effect. Consequently, since there was heterogeneity between the original studies (*I*^2^ = 93.5%, *p* < 0.001), a random-effect model was needed. The presence of publication bias was evaluated by using funnel plot test. Besides, study bias was evaluated using Egger’s test. Moreover, in this study, forest plots were used to estimate pooled effect size and effect of each study with their confidence interval (CI) to provide a visual image of the data. Pooled meta-logistic regression was used to present the pooled prevalence with a 95% confidence interval. Besides, subgroup and sensitivity analyses were employed.

## Results

### Characteristics of the included studies

A total of 840 potential articles were identified through the systematic literature search. After removal of duplicates, 413 articles were screened by title and abstract, and 74 were found to be eligible for full-text assessment. Of these full-text-screened articles, 38 (including 19,803 study participants) were found to be eligible for meta-analysis. Table 2 shows the general characteristics and outcomes of included studies.

**Table 2:** General characteristics and outcomes of the included studies (*n* = 38)

No	Author and year	Country	Sample size	Study design	*P* (%)	Shypo	Hypo	Shyper	Hyper	Study quality
1	Zhu, Y. et al. (2019) [26]	China	1677	Cross sectional	23.79	4.89	9.3	1.13	3.16	Good
2	Vamshidhar et al. (2020) [16]	India	50	Cross sectional	16	8	6	0	2	Good
3	Tudor, R. M. et al. (2018) [10]	Ireland	618	Cohort	30.5	15.5	3.5	9.7	1.8	Good
4	Subekti, I. et al. (2017) [11]	Indonesia	303	Cross sectional	9.9	0	7.59	0	2.31	Good
5	Papazafiropoulou, A. et al. (2010) [27]	Greek	1092	Cross sectional	12.3					Good
6	Palma, C. C. et al. (2013) [22]	Brazil	304	Cross sectional	13.3	12	0.7	0.3	0.3	Good
7	Ogbonna, S. U. et al. (2019) [28]	Nigeria	354	Cross sectional	12.4	0	11.6	0	0.8	Good
8	Khatiwada, S. et al. (2015) [29]	Nepal	401	Cross sectional	35.4	0	31.17	0	4.05	Good
9	Khassawneh, A. H. et al. (2020) [42]	Jordan	998	Case control	26.7	12.5	9.5	2.3	2.3	Good
10	Díez, J. J. et al. (2011) [7]	India	75	Cross sectional	25.2	14.6	8	1.3	1.3	Good
11	Demitrost, L. et al. (2012) [48]	India	202	Retrospective study	31.2	16.3	11.4	2	1.5	Good
12	Al-Wazzan, H. et al. (2010) [36]	Kuwait	1580	CS and CC	12.9	45.1	20.1	13.2	5.9	Good
13	Al-Geffari, M. et al. (2013) [23]	Saudi	411	Cross sectional	28.5	9.5	15.8	2.7	0.5	Good
14	Radaideh, A. et al. (2004) [30]	Jordan	908	Cross sectional	12.5	4	6.2	1.2	4	Good
15	Vikhe, V. B. et al. (2013) [9]	India	50	Case control	30	14	22	6	2	Good
16	Yadav, A. et al. (2021) [47]	India	2219	Cohort	20.3	14.1	3.2	0	3	Good
17	Peters, K. E. et al. (2020) [46]	Australia	1408	Cohort	16.8					Good
18	Ozair, M. et al. (2021) [31]	India	250	Cross-sectional	28	18.8	8	0	1.2	Good
19	Elgazar, E. H. et al. (2019) [32]	Egypt	200	Cross sectional	29	13	7	3	6	Good
20	Moslem, F. et al. (2015) [14]	Bangladesh	232	Cross sectional	10					Good
21	Sarfo-Kantanka O. et al. (2017) [43]	Ghana	302	Case control	12.2					Good
22	Ghazali S. M. et al. (2010) [40]	Nigeria	64	Case control	29.7	15.8	0	5.2	0	Good
23	Jalal M. J. et al. (2019) [39]	India	50	Case control	16	10	6	0	0	Good
24	Akbar D. H. et al. (2006) [41]	Saudi	74	Case control	9.5	6.75	2.7	0	0	Good
25	Hussain T. et al. (2019) [45]	India	700	Prospective	21.7	8	9.3	2.1	2.3	Good
26	Younes Y. R. (2020) [33]	Iraq	90	Cross sectional	17.7	11.1	3.3	1.1	2.2	Good
27	Jugati A., Biradar M. (2018) [51]	India	200	Case series	25	13.5	5.5	2.5	3.5	Good
28	Haque S. I. U. et al. (2019) [34]	Pakistan	334	Cross sectional	30.3	15.6	2.7	10.2	1.8	Good
29	Elmenshawi I. et al. (2017) [49]	Egypt	100	Retrospective study	29	23	3	0	3	Good
30	Al-Lehibi K. I. et al. (2019) [50]	Iraq	1341	Retrospective study	8	0	7	0	1	Good
31	Noori D. A. S. et al. (2019) [38]	Iraq	75	Case control	13.3	4	6.6	1.3	1.3	Good
32	Ahmed A. A. et al. (2017) [3]	Libya	369	Cross sectional	9.5	5	2.3	0	2.2	Good
33	Papanna J. et al. (2019) [8]	India	100	Cross sectional	32	18	8	1	5	Good
34	Mausam Jain et al. (2020) [35]	India	263	Cross sectional	25.44	5.7	14.8	1.9	3.04	Good
35	Jain G. et al. (2013) [37]	India	200	Cross sectional	16	7.5	4.5	1.5	2.5	Good
36	Padma V. et al. (2015) [44]	India	100	Case control	29	16	10	2	1	Good
37	Vij V. et al. (2014) [53]	India	40	Case control	25	15	10	0	0	Good
38	Aljabri K. S. (2019) [52]	Saudi	2069	Cross-sectional	20.7	3	11.1	6	1.6	Good

*Shypo* subclinical hypothyroidism, *Hypo* hypothyroidism, *Shyper* subclinical hyperthyroidism, *Hyper* hyperthyroidism, *CS* and *CC* cross sectional and case control

The studies that are included were done all over the globe and published between 2000 and 2022. The meta-analysis included 38 studies that revealed the prevalence and contributing factors of TD among T2DM patients. Among all the papers, 6 of them were from Africa [3, 31, 35, 43, 45, 51], 28 were from Asia [3, 7–9, 11, 14, 16, 23, 24, 29, 32–34, 37–42, 44, 46, 47, 49, 50, 52–58], 1 was from Australia [48], 2 were from Europe [10, 30], and 1 was from South America [22]. Regarding the study design, 9 were case–control, 1 case series, 3 were cohort, 21 were cross-sectional, 1 prospective, and 3 retrospective studies. The minimum sample size was 40 participants in a case–control study conducted in India [55], while the highest sample size was 2219 participants, in a cohort study conducted in India [49].

The Joana JBI standardized critical appraisal instrument for prevalence studies indicated that none of the included studies was of poor quality. After quality assessment, the 38 studies were subjected to meta-analysis. Table 2 presents the characteristics and outcomes of the reviewed studies. The prevalence of TD was estimated based on measurement of blood levels of TSH, FT3, and FT4 among the T2DM patients from all over the world.

### Prevalence of TD among adult T2DM patients

Thirty-eight published studies were included in this systematic review and meta-analysis, and all of these studies were used to estimate the pooled prevalence of TD among T2DM patients.

**Fig. 2:**
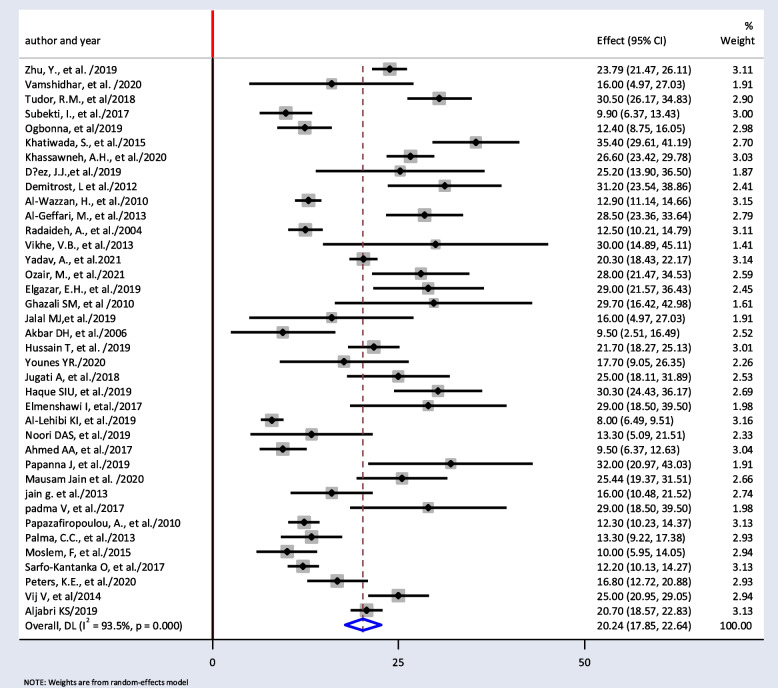
Pooled prevalence of thyroid dysfunction among T2DM patients from random effect model

The minimum prevalence of TD was 8% from a retrospective study done in India [52], and the maximum prevalence of TD was found to be 35.4% in Nepal [32]. The *I*^2^ test result showed high heterogeneity (*I*^2^ = 93.5%, = 0.000). The pooled prevalence of TD among T2DM was found to be 20.24% (95% *CI*: 17.85, 22.64) using random-effect model (Fig. 2).

### Prevalence of types of TDs and subgroup analysis

Thirty-four papers were used to estimate the pooled prevalence of subgroups of TD. The pooled prevalence of subclinical hypothyroidism, hypothyroidism, subclinical hyperthyroidism, and hyperthyroidism were found to be 11.87% (95% *CI*: 6.90, 16.84), 7.75% (95% *CI*: 5.71, 9.79), 2.49% (95% *CI*: 0.73, 4.25), and 2.51% (95% *CI*: 1.89, 3.13), respectively.

Subgroup analysis based on the study design showed that the weighted pooled prevalence of TD was 18.97% (95% *CI*: 15.93, 22.01), 22.17% (95% *CI*: 16.41, 27.92), 21.32% (95% *CI*: 14.37, 28.27), 22.33% (95% *CI*: 4.34, 40.32), 21.70% (95% *CI*: 18.27, 25.13), and 25% (95% *CI*: 18.11, 31.89) among the cross-sectional, cohort, case control, retrospective, prospective, and case series respectively. Table 3 shows the summary of the subgroup analysis of studies.

**Table 3:** Summary of sub-group analysis of studies included in the meta-analysis on the prevalence and associated factors of TD among T2DM patients

Subgroup	Random effect (95% CI)	Test of heterogeneity (*I*^2^)
**By types of TD**		
Subclinical hypothyroidism	11.87% (95% *CI*: 6.90, 16.84)	98.3%
Hypothyroidism	7.75% (95% *CI*: 5.71, 9.79)	88.2%
Subclinical hyperthyroidism	2.49% (95% *CI*: 0.73, 4.25)	83.4%
Clinical hyperthyroidism	2.51% (95% *CI*: 1.89, 3.13)	0.00%
**By study design**		
Cross-sectional	19.40% (95% *CI*: 16.40, 22.40)	92.6%
Cohort	22.41% (95% *CI*:15.82, 29.01)	91.3%
Case control	20.55% (95% *CI*: 14.50, 26.60)	90.7%
Retrospective	22.33% (95% *CI*: 4.34, 40.32)	95.8%
**By continent**		
Asia	21% (95% *CI*: 18.06, 23.94)	94.%
Europe	21.30% (95% *CI*: 3.46, 39.13)	98.2%
Africa	18.42% (95% *CI*: 12.46, 24.39)	86.8%
**By year**		
2006–2010	14.61% (95% *CI*: 7.31, 21.90)	72.2%
2011–2015	20.24% (95% *CI*: 13.90, 26.58)	92.6%
2016–2022	21.08% (95% *CI*: 17.84, 24.32)	94.0%

**Fig. 3:**
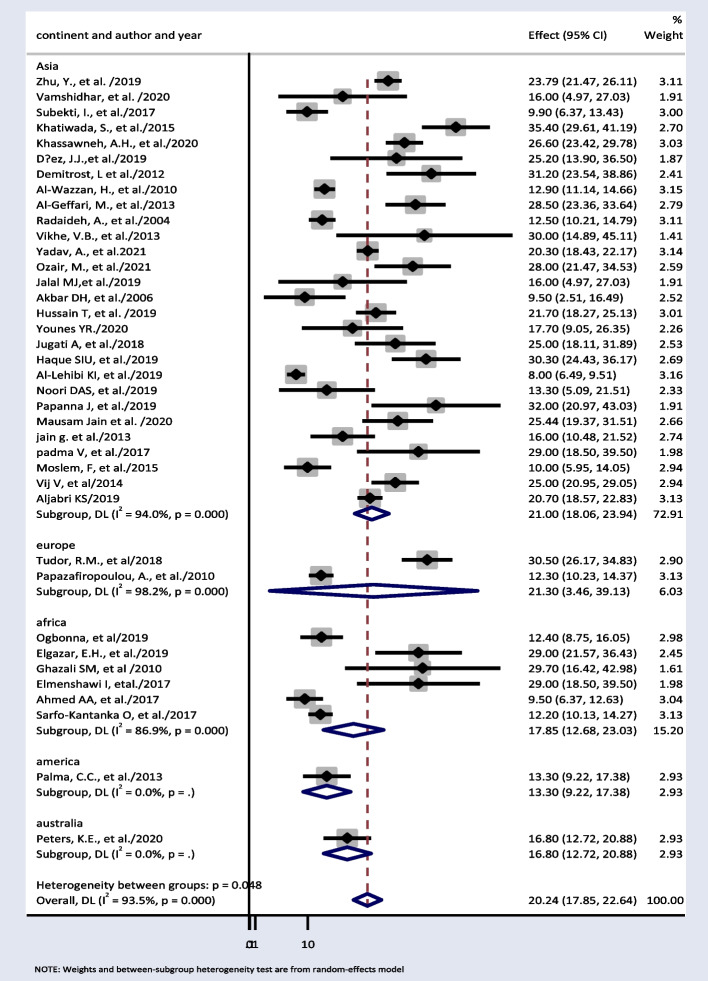
Pooled prevalence of thyroid dysfunction based on different continents

Thirty-eight papers were used to estimate subgroup analysis based on continent. The result showed weighted pooled prevalence of TD was 21% (95% *CI*: 18.06, 23.94), 21.30% (95% *CI*: 3.46, 39.13), and 17.85% (95% *CI*: 12.68, 23.03) in Asia, Europe, and Africa, respectively (Fig. 3).

### Factors associated with TD among T2DM patients

In this meta-analysis, seven studies were included to examine the factors associated with TD among T2DM [30–32, 39, 44, 52, 59]. Being female [23, 30–32, 39, 44, 59], central obesity [31], *HbA1c* ≥ 7% [31, 44], > 5-year duration of DM [31, 44, 59], educational level [59], diabetic neuropathy and retinopathy [31, 59], family history of TD [23, 32], and smoking [32, 39, 44] were found to be associated with T2DM. Tables 4 and 5 shows summary statistics of the risk factors.

**Table 4:** Summary statistics for the prevalence of TD by risk factors for each study

Associated factors		Authors and year						
		**Ogbonna S. et al. (2019) **[28]	**Yimer, R. M. et al. (2022) **[2]	**Papazafiropoulou, A. et al. (2010) **[27]	**Khatiwada, S. et al. (2015) **[29]	**Khassawneh, A. et al. (2020) **[42]	**AI Wazzan, H. et al. (2010) **[36]	**AI-Geffari, M. et al. (2013) **[23]
**Sex**	**Female**	56.5%	78.2%	78.4%	46.77%	47.9%	67.2%	68.6%
	**Male**	43.5%	21.8%	21.6%	53.23%	52.1%	32.8%	31.4%
**Central obesity**	**Yes**	30%	-	-		-	-	
	**No**	14%	-	-		-	-	
**HbA1c**	** ≥ 7%**	27%	-	-		35.2%	-	
	** < 7%**	17%	-	-		21.8%	-	
**Duration of DM**	** < 5 years**	34%	58.6%	-		-	-	17.3%
	** ≥ 5 years**	10%	41.4%	-		-	-	
**Educational level**	**No**	-	27.3%	-		-	-	
	**1°**	-	36.9%	-		-	-	
	**2°**	-	30.4%	-		-	-	
	**Post 2°**	-	5.4%	-		-	-	
**Diabetic neuropathy**	**Yes**	17%	50%	-		-		
	**No**	27%	50%	-			-	
**Diabetic retinopathy**	**Yes**	-	29.3%	-			-	
	**No**	-	70.4%	-			-	
**Family history of TD**	**Yes**	-	-	-	20.8%		-	14.7%
	**No**	-	-	-			-	
**Smoking**	**Yes**	-	-	-	19.3%	15%	12.7%	
	**No**	-	-	-		75.2%	11.8%	
	**Ex**					9.8%	75.5%

**Table 5:** Summary statistics for risk factors of TD among T2DM patients

Associated factors		Author and year						
		**Ogbonna S. et al. (2019) **[28]	**Yimer, R. M. et al. (2022) **[2]	**Papazafiropoulou, A. et al. (2010) **[27]	**Khatiwada, S. et al. (2015) **[29]	**Khassawneh, A. et al. (2020) **[42]	**AI Wazzan, H. et al. (2010) **[36]	**AI-Geffari, M. et al. (2013) **[23]
Female gender	AOR	3.8	2.5	0.222	1.44	1.76	1.7	1.95
	95% CI	(1.7–8.4)	(1.15–5.67)	(0.14–0.35)	1.09–1.91	1.12–2.75	1.2–2.9	1.36–2.78
	*p*-value	0.002	0.022	< 0.001	0.01	0.013	< 0.001	< 0.0001
The presence of central obesity	AOR	2.5	-	-	-	-	-	-
	95% CI	(1.2–5.2)	-	-	-	-	-	-
	*p*-value	0.001	-	-	-	-	-	-
HBA1C ≥ 7%	AOR	4.3	-	-	-	2.55	-	-
	95% CI	(2.1–8.9)	-	-	-	1.45–4.43	-	-
	*p*-value	0.025	-	-	-	0.001	-	-
Duration of DM ≥ 5 years	AOR	3.3	0.29	-	-	-	-	1.66
	95% CI	(1.5–7.9)	(.092–0.95)	-	-	-	-	1.06–2.61
	*p*-value	0.012	0.04	-	-	-	-	< 0.0001
The presence of diabetic neuropathy	AOR	4.8	3.3	-	-	-	-	-
	95% CI	(2.2–10.5)	(1.19, 8.92)	-	-	-	-	-
	*p*-value	0.001	0.021	-	-	-	-	-
The presence of diabetic retinopathy	AOR	-	9.3	-	-	-		-
	95% CI	-	(2.05, 42.51)	-	-	-	-	-
	*p*-value	-	0.04	-	-	-	-	-
Family history of TD	AOR	-	-	-	2.57	-	-	-
	95% CI	-	-	-	2–3.31	-	-	3.39
	*p*-value	-	-	-	< 0.001	-	-	2.47–4.63
Smoking	AOR	-	-	-	2.56	-	7.8	< 0.0001
	95%CI	-	-	-	1.99–3.29	-	1.2–2.9	-
	*p*-value	-	-	-	< 0.001	-	< 0.001	-
Age > 50	AOR	-	-	-	-	3.9	-	-
	95% CI	-	-	-	-	2.15–7.05	-	-
	*p*-value	-	-	-	-	< 0.001	-	-

### Sensitivity test

**Fig. 4:**
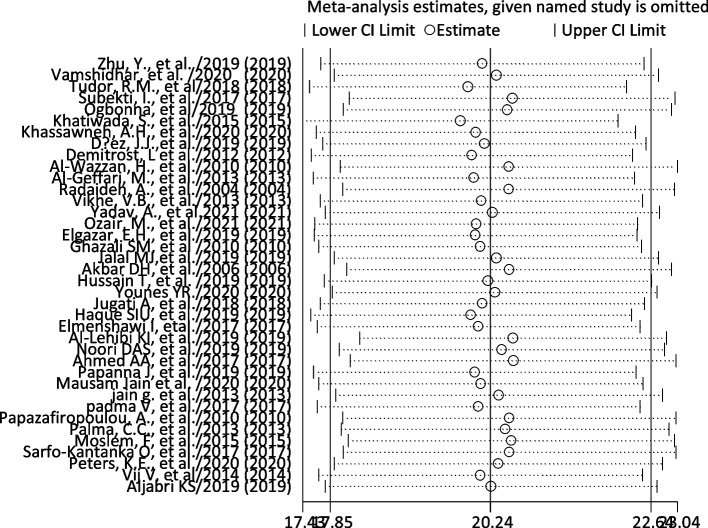
Sensitivity analysis of pooled prevalence of thyroid dysfunction for each study being removed one at a time

We did the sensitivity analysis of the prevalence of TD among T2DM by applying a random-effects model (Table 6). The analysis was done to evaluate the effect of each study on the pooled estimated prevalence of TD by excluding each study step by step. The result showed that excluded studies did not show a significant difference in the prevalence of TD among T2DM (Fig. 4 and Table 6).

**Table 6:** Sensitivity analysis of included studies to estimate the pooled prevalence of thyroid dysfunction among type 2 diabetes mellitus patients

Study omitted	Estimate	95% confidence interval
Zhu, Y. et al. (2019) [26]	20.119591	17.704163, 22.535021
Vamshidhar et al. (2020) [16]	20.32708	17.904331, 22.749828
Tudor, R. M. et al. (2018) [10]	19.90391	17.535975, 22.271847
Subekti, I. et al. (2017) [11]	20.564457	18.126856, 23.00206
Ogbonna et al. (2019) [28]	20.492756	18.040697, 22.944815
Khatiwada, S. et al. (2015) [29]	19.787039	17.430199, 22.143877
Khassawneh, A. H. et al. (2020) [42]	20.020098	17.633699, 22.406496
Diez, J. J. et al. (2011) [7]	20.147766	17.730288, 22.565245
Demitrost, L. et al. (2012) [48]	19.961981	17.558344, 22.365618
Al-Wazzan, H. et al. (2010) [36]	20.517977	17.998659, 23.037292
Al-Geffari, M. et al. (2013) [23]	19.991449	17.590086, 22.392815
Radaideh, A. et al. (2004) [30]	20.51273	18.030352, 22.995108
Vikhe, V. B. et al. (2013) [9]	20.101665	17.692612, 22.510721
Yadav, A. et al. (2021) [47]	20.270761	17.772118, 22.769403
Ozair, M. et al. (2021) [31]	20.029493	17.617088, 22.441898
Elgazar, E. H. et al. (2019) [32]	20.016003	17.605022, 22.426983
Ghazali S. M. et al. (2010) [40]	20.085546	17.674978, 22.496117
Jalal M. J. et al. (2019) [39]	20.32708	17.904331, 22.749828
Akbar D. H. et al. (2006) [41]	20.520798	18.092993, 22.948603
Hussain T. et al. (2019) [45]	20.203516	17.758429, 22.648603
Younes Y. R. (2020) [33]	20.304136	17.875332, 22.732943
Jugati A. et al. (2018) [51]	20.117435	17.694298, 22.540575
Haque S. I. U. et al. (2019) [34]	19.949656	17.55298, 22.346331
Elmenshawi I. et al. (2017) [49]	20.062523	17.649525, 22.475519
Al-Lehibi K. I. et al. (2019) [50]	20.578279	18.284489, 22.872068
Noori D. A. S. et al. (2019) [38]	20.410748	17.981333, 22.84016
Ahmed A. A. et al. (2017) [3]	20.58	18.145374, 23.014626
Papanna J. et al. (2019) [8]	20.009239	17.601709, 22.416771
Mausam Jain et al. (2020) [35]	20.097679	17.675913, 22.519445
Jain G. et al. (2013) [37]	20.368254	17.92613, 22.810379
Padma V. et al. (2015) [46]	20.062523	17.649525, 22.475519
Papazafiropoulou, A. et al. (2010) [27]	20.523239	18.034031, 23.012445
Palma, C. C. et al. (2013) [22]	20.461063	18.010532, 22.911594
Moslem, F. et al. (2015) [14]	20.554682	18.118074, 22.991287
Sarfo-Kantanka O. et al. (2017) [43]	20.525778	18.038151, 23.013405
Peters, K. E. et al. (2020) [46]	20.357279	17.902861, 22.811699

### Publication bias

**Fig. 5:**
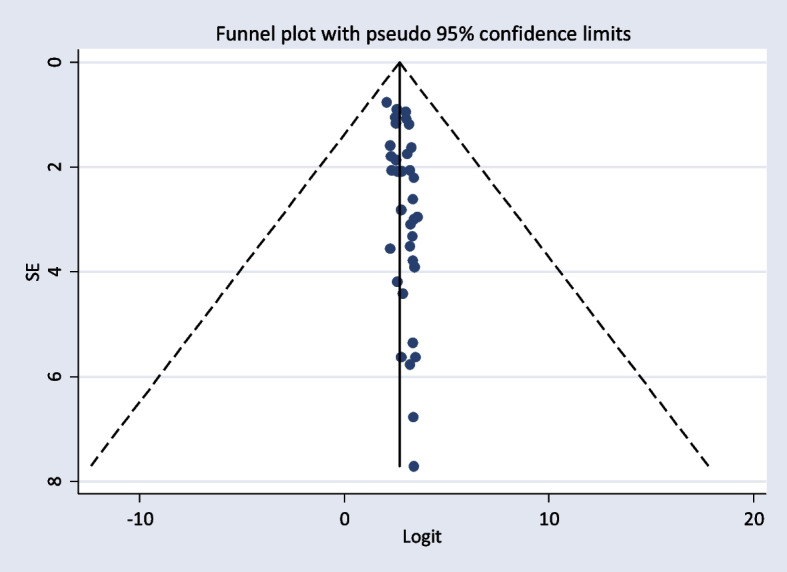
Funnel plot showing publication bias

The included studies were assessed for potential publication bias visually by funnel plot. The funnel plot was asymmetrical which indicate the presence of publication bias (Figs. 4 and 5). Besides, the result of Egger’s test indicated there was publication bias, *P*-value < 0.05. The *P*-value was found to be 0.019 (Table 7).

**Table 7:** Egger’s test showing publication bias

Std_eff	Coef	Std. err	*T*	*P* > t	[95% *CI*]
Slope	2.419087	0.11522	21.00	0.000	(2.18541, 2.652764)
Bias	0.1914215	.0654948	2.92	0.006	(.0585919, 0.3242511)

**Fig. 6:**
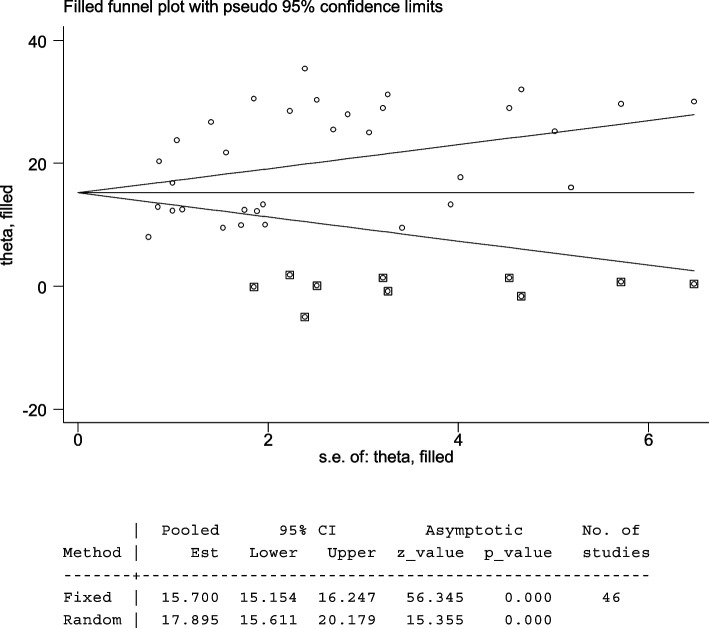
Trim-and-fill analysis of TD among T2DM patients

The Egger’s test indicated that the unpublished findings might have shown a lower magnitude of TD. Adjusting the findings using the trim-and-fill method would provide a bias-adjusted effect estimate. Therefore, to do so, a trim-and-fill method analysis was conducted. A bias-adjusted effect estimate of TD showed 17.89 (15.611, 20.179), assuming there are missing studies (Fig. 6).

### Meta-regression

Meta-regression was performed to determine the source of heterogeneity by considering sample size and year of publication as a covariate. There was no significant relationship between the year of publication and the prevalence of TD. In addition to this, meta-regression was also conducted that explains the linear prediction of the prevalence of TD and function of sample size. Similarly, there was no significant relationship between the sample size and the prevalence of TD (Table 8).

**Table 8:** Meta-regression based on publication year and sample size

Heterogeneity source	Coefficient	Standard error	*p*-value
Publication year	0.2935312	0.5478136	0.595
Sample size	0.0012641	0.0045391	0.782

## Discussion

The pooled prevalence of TD in this systematic review and meta-analysis was found to be 20.24% (95% *CI*: 17.85, 22.64). Funnel plots and Egger’s tests showed there was publication bias among included studies. Trim-and-fill method was used to correct the results. A bias-adjusted effect estimate of TD showed 17.89 (15.611, 20.179), assuming there are missing studies. This result was higher than the 11.7% seen in a Colorado TD survey of 25,862 people who attended a state health fair [12]. It was also higher than the National Health and Nutrition Examination Survey (NHANES III Study), a survey of 17,353 subjects (5.9%) [60]. Type 2 diabetic patients have a higher prevalence of TD than non-diabetics; T2DM lowers TSH levels and impairs the conversion of T4 to T3 in the peripheral tissues. Poorly managed T2DM can lead to insulin resistance and hyperinsulinemia. This, in turn, promotes the growth of thyroid tissue and increases the formation of nodules and the size of goiters. In addition, while metformin can be beneficial in both T2DM and TD patients, there are some other antidiabetic drugs like sulfonylureas, and thiazolidinedione group drugs like pioglitazone can negatively impact thyroid function [12].

The most common type of TD seen in this systematic review and meta-analysis was subclinical hypothyroidism (11.87%, 95% *CI*: 6.90, 16.84). This result was in line with a systematic review and meta-analysis done globally (12% (95% *CI*: 10%, 14%) [61] and higher than results reported in general population (4–9%) [1]. More than half of TDs reported are undiagnosed or subclinical because symptoms of TD are easily mistaken for depression, menopause, or obesity [62]. The presence of subclinical hypothyroidism may increase cardiovascular risk by aggravating dyslipidemia, insulin resistance, obesity, and vascular endothelial dysfunction [1, 63].

Hypothyroidism was the second most common form of TD found in this systematic review and meta-analysis (7.75% (95% *CI*: 5.71, 9.79). This finding was also higher than that of the NHANES III Study (4.6%) [1]. Overall hypothyroidism is the most common type of TD among T2DM patients. Worldwide, environmental iodine deficiency is the most common cause of hypothyroidism [64]. Globally, more than 1.9 billion individuals have inadequate iodine nutrition Despite the implementation of iodine supplementation programs (e.g., salt iodization), iodine intake remains suboptimal in large parts of the world [64, 65].

In this review, the pooled prevalence of hyperthyroidism was 2.51% (95% *CI*: 1.89, 3.13). This was similar to the community-based study done in Wickham among 2779 participants (2%) [66].

Subgroup analysis based on continent showed 21% (95% *CI*: 18.06, 23.94) and 17.85% (95% *CI*: 12.68, 23.03) pooled prevalence of TD in Asia and Africa respectively. TDs have been documented in more than 110 countries, the most of which are in Africa, Asia, and Latin America [67]. In comparison with other continents, this result is high. This is because, in the developed world, the frequency of undiagnosed TD is anticipated to be declining as a result of extensive thyroid function testing and low treatment initiation thresholds. However, in continents such as Africa and Asia, this is challenge [68]. Iodine deficiency is a major public health problem throughout Africa and is the commonest cause of TDs in this continent [69]. At least 350 million Africans are at risk of iodine deficiency. A total of 25% of the global burden of iodine deficiency occurs in Africa [70].

Seven studies were included to examine the factors associated with TD among T2DM [30–32, 39, 44, 52, 59], and different factors were found associated with TD. Among them, sex was found to be the prominent determinant of TD. All of the studies indicated a statistically significant association between sex and TD that shows a higher risk of TD with being female. In a cross-sectional research of 411 T2DM patients in Saudi Arabia, it was discovered that being female has 1.95 higher odds of having TD as compared with males (*OR* = 1.95, 95% *CI*: 1.36–2.78, *p* = 0.0001) [23]. Female gender was also a risk factor for TD, according to a study conducted in Greece among 1092 T2DM patients (*OR* = 0.222, 95% *CI* = 0.141–0.352, *p* = 0.001) [30]. These results were also similar to the study conducted in Nepal and Kuwait (*RR* = 1.44, 95% *CI* = 1.09–1.91, *p* = 0.01) and (*OR* = 1.7, 95% *CI*: 1.2–2.9, *p* =  < 0.001) respectively [32, 39].

In a research done in Nigeria among 354 T2DM patients, it was found females who had T2DM were 3.8 times more likely to develop TD than their male counterparts (*OR* = 3.8, 95% *CI* = 1.7–8.4, *p* = 0.002) [31]. Similar result was found in a case–control study conducted in Ethiopia. Being female had 2.5 times the odds of having TD than their male counterparts 2.5 (*OR* = 2.5, 95% *CI* = 1.15–5.67, *p* = 0.022) [59].

The prevalence of TD in diabetic patients is influenced by female gender in which T2DM patients who are female are more likely to develop TD. This in because sex hormones and the skewed inactivation of the X chromosome are suspected to be triggers for hypothyroidism and hyperthyroidism [71]. Another factor contributing to the high prevalence of TD in women is the interaction between TH and hormones that change during the menstrual cycle [72].

Smoking was also found associated with TD among T2DM patients [32, 39]. In the study conducted in Kuwait among 204 T2DM patients, ex-smokers and current smoker patients were more liable for TD (*OR* = 18.1, 95% *CI*: 10.1–32.5) and (*OR* = 7.8, 95% *CI*: 3.5–17.7) respectively [39]. Similar finding was also found in the study done in Nepal. Smokers had 2.32 higher odds of having TD (*OR* = 2.32, 95% *CI*: 1.85–2.91) [32].

The reason behind this is that cigarette smoke contains cyanide which is converted to thiocyanate, which disrupts iodine uptake and blocks the production of THs [73]. Many other components of cigarettes also have antithyroid effects, such as decreasing T3 receptor binding or post-receptor activities in the liver, muscle, or both. According to reports, smoking/nicotine causes an unnaturally high metabolism, masking the fatigue/lethargy associated with hypothyroidism. When the smoker quits, this masking is removed, and the full effects of hypothyroidism on the metabolism and thyroid are felt. And, for smokers with undiagnosed TD, without proper TH treatment, smoking cessation seems to double weight gain whammy, as they lose the appetite suppressant, metabolism-upping effects of nicotine, and experience the full effects of the hypothyroidism [39].

Besides, among the seven papers used to assess associated factors, two of them reported that TD is associated with *HbA1c* ≥ 7% [31, 44]. A study conducted in Nigeria found that T2DM patients with ≥ 7% HbA1c were 4.3 times more likely to develop TD than their counterparts with good glycemic control (H*bA1c* < 7%) (*OR* = 4.3, 95% *CI* = 2.1–8.9, *p* = 0.025) [31]. A case–control study conducted in Jordan was also in line with this study. It was found that patients who had *HBA1c* ≥ 7% were found to have 2.55 higher odds of having TD when compared with patients who have *HBA1c* ≤ 7% (*OR* = 2.55, 95% *CI* = 1.45–4.43, *p* = 0.001) [44]. The association of hyperglycemia with TD may be due to the adverse effects of chronic hyperglycemia on the hypothalamic-pituitary axis where it blunts or abolishes the nocturnal TSH peak [59].

It was also found central obesity (abnormal waist circumference) was significantly associated with TD in a case–control study done in Nigeria (*OR* = 2.5, 95% *CI* = 1.5–5.2, *p* = 0.001) [31]. Leptin is known to be an important neuroendocrine regulator of the hypothalamo-pituitary-thyroid axis by regulating TRH gene expression in the paraventricular nucleus. Iodine deficiency, autoimmune thyroiditis, and mutations in the TSH receptor genes are some of the other hypotheses put forward to explain the association between increasing TSH, obesity, and subclinical hypothyroidism in some populations [31].

Duration of diabetes was found to be associated with TD in two of the studies [23, 31]. In a research done in Nigeria among 354 T2DM patients, DM duration > 5 years (*OR* = 3.3, *p* = 0.012) was a risk factor for TD [31]. A cross-sectional study conducted in Saudi Arabia diabetes also reported that duration of more than 10 years has been shown to be an important risk factor (*OR* = 1.66, 95% *CI*: 1.06–2.61) [23]. This could indicate that the duration of diabetes mellitus (DM) is a risk factor for the development of TD, as persistent hyperglycemia inhibits the peripheral deiodination of T4 to T3, resulting in TD [31].

Educational level was also found associated with TD. It was found T2DM patients who attend primary school had 1.5 higher odds of having TD (*OR* = 1.5, 95% *CI*: 1.03–1.67). On the other hand, T2DM patients who have secondary education and post-secondary education had less likely to have TD (*OR* = 0.11, 95% *CI* = 0.06–0.48, *p* = 0.02) and (*OR* = 0.21, 95% *CI* = 0.062–0.85, *p* = 0.028); this implies better educational levels being protective. This is logical because a higher educational level is linked to improved blood glucose control, which is linked to good thyroid function [59].

Among the seven papers used to assess associated factors, two studies showed that previous family history of TD was associated with TD. A cross-sectional study conducted in Saudi Arabia among 411 T2DM patients found that diabetic patients with a positive family history of TD had a higher chance of developing TD (*OR* = 3.39, 95% *CI*: 2.47–4.63, *p* =  < 0.0001) [23]. A study from Nepal also having previous family history of TD increased the risk by 2.57 (*RR* = 2.57, 95% *CI* = 2–3.31, *p* < 0.001) [32].

Age > 50 was significant factor with OR of 3.9 (95% *CI* 2.151–7.052, *p* < 0.001). This can be explained by that elderly patients might have had undetected diabetes for a longer time [44]. About the factor presence of retinopathy [59] with an odds ratio of 9.3 (95% *CI*: 2.05–42.51, *p* =0.04), and for factor presence of neuropathy [59] with an odds ratio of (*OR* =3.3, 95% *CI* =1.19–8.92, *p* =0.021) [32] showed the presence of retinopathy and neuropathy were risk factors of TD among T2DM patients respectively [39].

## Conclusion and recommendation

The current systematic review and meta-analysis showed that the pooled prevalence of TD among T2DM patients was found to be higher compared with the general population. The pooled prevalence of TD among T2DM was found to be 20.24% (95% *CI*: 17.85, 22.64) using random-effect model. The pooled prevalence of subclinical hypothyroidism, hypothyroidism, subclinical hyperthyroidism, and hyperthyroidism was found to be 11.87% (95% *CI*: 6.90, 16.84), 7.75% (95% *CI*: 5.71, 9.79), 2.49% (95% *CI*: 0.73, 4.25), and 2.51% (95% *CI*: 1.89, 3.13), respectively. Being female, obesity, family history of TD, smoking, advanced age, and family history of DM were factors associated with TD among adult T2DM patients.

We recommend it is important to screen for TD in T2DM patients as each of these endocrinopathies and their complex interdependent interactions increase cardiovascular risks.

## Strength and limitations

This systematic review and meta-analysis revealed the pooled figure on prevalence of TD, its subtypes, and associated factors of TD among T2DM patients. This will give researchers, policymakers, and public health stakeholders the empirical knowledge they need to develop health-promoting policies, allocate resources, and set priorities for monitoring future trends.

The limitations of this systematic review and meta-analysis is that the search strategy was limited only to published articles, but unpublished papers may be missed. Only free online databases were used. In addition to this, only papers written in English were included. Time barrier was also one of the limitations. Moreover, it is essential to highlight that the paucity of research conducted in the area of thyroid dysfunction among type 2 diabetes patients in Europe, America, and Australia resulted in a restricted number of articles being incorporated into our analysis.

## Data Availability

The main part of the data generated or analyzed during this study is included in this published article. Other data will be available from the corresponding author upon request.

## Abbreviations

BMI: Body Mass Index
DM: Diabetes Mellitus
FT3: Free Triiodothyronine
FT4: Free Thyroxine
HbA1C: Hemoglobin A1C
IDF: International Diabetes Federation
mIU/L: Milli-international Unit per Liter
NHANES: National Health and Nutrition Examination Survey
pmol/L: Picomoles per Liter
PRISMA: Preferred Reporting Items for Systematic Reviews and Meta-Analyses
TD: Thyroid Dysfunction
T2DM: Type 2 Diabetes Mellitus
TH: Thyroid Hormone
TRH: Thyrotropin-releasing Hormone
TSH: Thyroid-stimulating Hormone

## References

[1] Wang C (2013). The Relationship between type 2 diabetes mellitus and related thyroid diseases. J Diabetes Res.

[2] Laulund AS, Nybo M, Brix TH, Abrahamsen B, Jørgensen HL, Hegedüs L (2014). Duration of thyroid dysfunction correlates with all-cause mortality The OPENTHYRO Register Cohort. PloS one.

[3] Ahmed AA, Mohamed SB, Elmadi SA, Abdorabo AA, Ismail IM, Ismail AM (2017). Assessment of thyroid dysfunctions in type 2 diabetes mellitus patients in Surman, Western-Libya. Int J Clin Exp Med Sci.

[4] DeFronzo RA, Ferrannini E, Groop L, Henry RR, Herman WH, Holst JJ (2015). Type 2 diabetes mellitus. Nat Rev Dis Primers.

[5] Inzucchi SE, Sherwin RS (2011). Type 2 diabetes mellitus.

[6] Olokoba AB, Obateru OA, Olokoba LB (2012). Type 2 diabetes mellitus: a review of current trends. Oman Med J.

[7] Diez JJ, Sánchez P, Iglesias P. Prevalence of thyroid dysfunction in patients with type 2 diabetes. Exp Clin Endocrinol Diabetes. 2011;119(04):201–7. 10.1055/s-0031-1271691 21465427

[8] Papanna J, Bettegowda S (2019). Thyroid dysfunction among type 2 diabetes mellitus patients: a study from rural hospital. J Med Sci Clin Res.

[9] Vikhe VB, Kanitkar SA, Tamakuwala KK, Gaikwad AN, Kalyan M, Agarwal RR (2013). Thyroid dysfunction in patients with type 2 diabetes mellitus at tertiary care centre. Natl J Med Res.

[10] Tudor RM, Garrahy A, Woods CP, Crowley RK, Tormey WT, Smith D (2020). The prevalence and incidence of thyroid dysfunction in patients with diabetes - a longitudinal follow-up study. Ir J Med Sci.

[11] Subekti I, Pramono LA, Dewiasty E, Harbuwono DS (2017). Thyroid dysfunction in type 2 diabetes mellitus patients. Acta Med Indones.

[12] Kalra S, Aggarwal S, Khandelwal D (2019). Thyroid dysfunction and type 2 diabetes mellitus: screening strategies and implications for management. Diabetes Ther..

[13] Datchinamoorthi S, Rathanavel N, Rajagopalan B, Vanaja R (2016). Study of thyroid dysfunction in type II diabetes mellitus. Int J Pharm Sci Res.

[14] Moslem F, Bithi TS, Biswas A (2015). Prevalence of thyroid dysfunction among type-2 diabetes patients in an urban diabetes hospital. Bangladesh Open Sci J Clin Med.

[15] Biondi B, Kahaly GJ, Robertson RP (2019). Thyroid dysfunction and diabetes mellitus: two closely associated disorders. Endocr Rev.

[16] Vamshidhar IS, Rani SSS (2020). A study of association of thyroid dysfunctions in patients with type 2 diabetes mellitus. Maedica.

[17] Hage M, Zantout MS, Azar ST. Thyroid Disorders and Diabetes Mellitus. J Thyroid Res. 2011;2011:439463, 7:1–7. 10.4061/2011/439463 PMC3139205 21785689

[18] Ward RJ, Heald AH, Ogunmekan S, Fryer AA, Duff CJ (2018). Should we be screening for thyroid dysfunction in patients with type 2 diabetes mellitus?. Br J Gen Pract.

[19] Mohammed Hussein SM, AbdElmageed RM (2021). The relationship between type 2 diabetes mellitus and related thyroid diseases. Cureus.

[20] Díez JJ, Iglesias P (2012). Subclinical hyperthyroidism in patients with type 2 diabetes. Endocrine.

[21] Vondra K, Vrbikova J, Dvorakova K (2005). Thyroid gland diseases in adult patients with diabetes mellitus. Minerva Endocrinol.

[22] Palma CC, Pavesi M, Nogueira VG, Clemente EL, Vasconcellos Mde F, Pereira LCJ (2013). Prevalence of thyroid dysfunction in patients with diabetes mellitus. Diabetol Metab Syndr.

[23] Al-Geffari M, Ahmad NA, Al-Sharqawi AH, Youssef AM, AlNaqeb D, Al-Rubeaan K (2013). Risk factors for thyroid dysfunction among type 2 diabetic patients in a highly diabetes mellitus prevalent society. Int J Endocrinol..

[24] Akbar D, Ahmed M, Al-Mughales J (2006). Thyroid dysfunction and thyroid autoimmunity in Saudi type 2 diabetics. Acta Diabetol.

[25] Gaitonde DY, Rowley KD, Sweeney LB (2012). Hypothyroidism: an update. S Afr Fam Pract.

[26] Reid JR, Wheeler SF (2005). Hyperthyroidism: diagnosis and treatment. Am Fam Physician.

[27] World Health Organization. Definition and diagnosis of diabetes mellitus and intermediate hyperglycaemia: report of a WHO/IDF consultation.

[28] Shamseer L, Moher D, Clarke M, Ghersi D, Liberati A, Petticrew M (2015). Preferred Reporting Items for Systematic Review and Meta-Analysis Protocols (PRISMA-P) 2015: elaboration and explanation. BMJ..

[29] Zhu Y, Xu F, Shen J, Liu Y, Bi C, Liu J (2019). Prevalence of thyroid dysfunction in older Chinese patients with type 2 diabetes-a multicenter cross-sectional observational study across China. PLoS ONE.

[30] Papazafiropoulou A, Sotiropoulos A, Kokolaki A, Kardara M, Stamataki P, Pappas S (2010). Prevalence of thyroid dysfunction among Greek type 2 diabetic patients attending an outpatient clinic. J Clin Med Res.

[31] Ogbonna SU, Ezeani IU (2019). Risk factors of thyroid dysfunction in patients with type 2 diabetes mellitus. Front Endocrinol.

[32] Khatiwada S, Kc R, Sah SK, Khan SA, Chaudhari RK, Baral N (2015). Thyroid dysfunction and associated risk factors among Nepalese diabetes mellitus patients. Int J Endocrinol.

[33] Radaideh A, Mo M, Amari FL, Bateiha AE, El-Khateeb M, Naser P (2004). Diabetes mellitus in Jordan. Saudi Med J.

[34] Ozair M, Noor S, Raghav A, Siddiqi SS, Chugtai AM, Ahmad J (2018). Prevalence of thyroid disorders in North Indian type 2 diabetic subjects: a cross sectional study. Diabetes Metab Syndr.

[35] Elgazar EH, Esheba NE, Shalaby SA, Mohamed WF (2019). Thyroid dysfunction prevalence and relation to glycemic control in patients with type 2 diabetes mellitus. Diabetes Metab Syndr.

[36] Younes YR (2020). The prevalence of thyroid dysfunction in patients with type 2 diabetes mellitus. Chronic Dis J.

[37] Haque SIU, Syed TM, Tahir A (2019). Burden of thyroid dysfunction in patients of type 2 diabetes mellitus. PAFMJ.

[38] Aljabri KS, Alnasser IM, Facharatz BS, Bokhari SA, Alshareef MA, Khan PM. The frequency of hypothyroidism in Saudi community-based hospital: A retrospective single centre study. Trends Diabetes Metab. 2019;2(1):1–4.

[39] Al-Wazzan H, Daban A, Askar R, El-Shazly M (2010). Prevalence and associated factors of thyroid dysfunction among type 2 diabetic patients. Kuwait Alexandria Journal of Medicine.

[40] Jain G, Marwaha TS, Khurana A, Dhoat PS. Prevalence of thyroid disorders in patients of type 2 diabetes mellitus. Int J Med Dent Sci. 2013;2(2):153–61.

[41] Nair A, Jayakumari C, Jabbar PK, Jayakumar RV, Raizada N, Gopi A (2018). Prevalence and associations of hypothyroidism in Indian patients with type 2 diabetes mellitus. J Thyroid Res.

[42] Jalal MJA, Riyas B, Kumar AP (2019). Thyroid dysfunction in patients with type-2 diabetes mellitus in Kerala: a case–control study. Thyroid Res Pract.

[43] Ghazali S, Abbiyesuku F (2010). Thyroid dysfunction in type 2 diabetics seen at the University College Hospital, Ibadan. Nigeria. Niger J Physiol Sci..

[44] Khassawneh AH, Al-Mistarehi AH, Zein Alaabdin AM, Khasawneh L, AlQuran TM, Kheirallah KA (2020). Prevalence and predictors of thyroid dysfunction among type 2 diabetic patients: a case-control study. Int J Gen Med.

[45] Sarfo-Kantanka O, Sarfo FS, Ansah EO, Yorke E, Akpalu J, Nkum BC (2017). Frequency and determinants of thyroid autoimmunity in Ghanaian type 2 diabetes patients: a case-control study. BMC Endocr Disord.

[46] Padma V, Anand NN. Prevalence of Thyroid Dysfunction in Type 2 Diabetic Patients. Int J Pharm Biochem Sci. 2015;6(3):289–94.

[47] Hussain T, Barik BS, Nayak AR, Das S, Khadanga UK, Yadav V (2019). Prevalence and predictors of thyroid dysfunction among patients with type 2 diabetes mellitus attending a tertiary care hospital in an urban area of Bhubaneswar, Odisha. Thyroid Res Pract.

[48] Peters KE, Chubb SAP, Bruce DG, Davis WA, Davis TME (2020). Prevalence and incidence of thyroid dysfunction in type 1 diabetes, type 2 diabetes and latent autoimmune diabetes of adults: the Fremantle Diabetes Study Phase II. Clin Endocrinol.

[49] Yadav A, Yadav GAM, Narsingrao KK, Nanda Kumar LG, Yadav GSN (2021). Prevalence of thyroid disorders among patients with diabetes in rural South India. Diabetes Metab Syndr.

[50] Demitrost L, Ranabir S (2012). Thyroid dysfunction in type 2 diabetes mellitus: a retrospective study. Indian J Endocrinol Metab.

[51] Elmenshawi I, Alotaibi S, Alazmi A, Alazmi A, Alruwaili F, Alazmi N (2017). Prevalence of thyroid dysfunction in diabetic patients. J Diabetes Metab Disord.

[52] Al-Lehibi KI, Abdulrahman MI, Albassam ENA-A (2019). Thyroid dysfunction in type 2 diabetic patients and the effect of diabetes duration and anti-glycemic medications on mean tsh and a1c levels: a retrospective study. Int J Med Res Health Sci.

[53] Jugati A, Biradar M (2018). Thyroid dysfunction in patients with type 2 diabetes mellitus in a tertiary care center of North Karnataka. Medica.

[54] Aljabri KS (2019). The prevalence of thyroid disorders in patients with type 2 diabetes mellitus in Saudi community based hospital. Curr Res Diabetes Obes J.

[55] Vij V, Chitnis P, Gupta VK (2012). Evaluation of thyroid dysfunction among type II diabetic patients. Ijpbs.

[56] Jali M, Kambar S, Jali SM, Pawar N, Nalawade P (2017). Prevalence of thyroid dysfunction among type 2 diabetes mellitus patients. Diabetes Metab Syndr.

[57] Zeru MA, Tesfa E, Mitiku AA, Seyoum A, Bokoro TA (2021). Prevalence and risk factors of type-2 diabetes mellitus in Ethiopia: systematic review and meta-analysis. Sci Rep.

[58] Zafar M, Shahid SM, Alshammari RF, Kausar MA, Ginawi TA, Hatim AW, Wadi AM, Ali H, Hamed AA, Al-zahrani MS, Hussain A. Association of thyroid disorders with diabetes: A cross-sectional study. Nus Biosci. 2022;14(2).

[59] Tekalign AM, Habte FB, Yimer RM. Determinants of Thyroid Dysfunction among Type 2 Diabetes Patients Attending Private Hospitals in Dire Dawa, Eastern Ethiopia. medRxiv. 2022:2022–02.

[60] Grassetto G, Rubello D (2008). Thyroid disorders and diabetes mellitus. Minerva Med.

[61] Han C, He X, Xia X, Li Y, Shi X, Shan Z (2015). Subclinical hypothyroidism and type 2 diabetes: a systematic review and meta-analysis. PLoS ONE.

[62] Moini J, Pereira K, Samsam M. Epidemiology of thyroid disorders. Elsevier; 2020 Jan 8. 75-85

[63] Joffe BI, Distiller LA (2014). Diabetes mellitus and hypothyroidism: strange bedfellows or mutual companions?. World J Diabetes.

[64] Chiovato L, Magri F, Carlé A (2019). Hypothyroidism in context: where we’ve been and where we’re going. Adv Ther.

[65] de Benoist B, Andersson M, Takkouche B, Egli I (2003). Prevalence of iodine deficiency worldwide. The Lancet.

[66] Tunbridge WM, Evered DC, Hall R, Appleton D, Brewis M, Clark F (1977). The spectrum of thyroid disease in a community: the Whickham survey. Clin Endocrinol.

[67] Alam Khan V, Khan MA, Akhtar S (2002). Thyroid disorders, etiology and prevalence. J Med Sci.

[68] Taylor PN, Albrecht D, Scholz A, Gutierrez-Buey G, Lazarus JH, Dayan CM (2018). Global epidemiology of hyperthyroidism and hypothyroidism. Nat Rev Endocrinol.

[69] Ogbera AO, Kuku SF (2011). Epidemiology of thyroid diseases in Africa. Indian journal of endocrinology and metabolism.

[70] Okosieme OE (2006). Impact of iodination on thyroid pathology in Africa. J R Soc Med.

[71] Libert C, Dejager L, Pinheiro I (2010). The X chromosome in immune functions: when a chromosome makes the difference. Nat Rev Immunol.

[72] Jacobson MH, Howards PP, Darrow LA, Meadows JW, Kesner JS, Spencer JB (2018). Thyroid hormones and menstrual cycle function in a longitudinal cohort of premenopausal women. Paediatr Perinat Epidemiol.

[73] Babić Leko M, Gunjača I, Pleić N, Zemunik T (2021). Environmental factors affecting thyroid-stimulating hormone and thyroid hormone levels. Int J Mol Sci.

